# Women’s knowledge, attitudes and practices related to mother-to-child transmission of HTLV-1 in Peru

**DOI:** 10.1371/journal.pgph.0007103

**Published:** 2026-08-27

**Authors:** Juan Macalupu, Francesca Ginocchio, Alejandra Paiva, Paloma Cusihuallpa, Diana Rodríguez-Hurtado, Fernando Mejía-Cordero, Elsa González-Lagos, Eduardo Gotuzzo

**Affiliations:** 1 Instituto de Medicina Tropical Alexander von Humboldt, Hospital Cayetano Heredia, Lima, Peru; 2 Facultad de Medicina, Universidad Peruana Cayetano Heredia, Lima, Peru; BRAC University Institute of Educational Development, BANGLADESH

## Abstract

Human T-cell Lymphotropic Virus type 1 (HTLV-1) can be transmitted from mother to child through breastfeeding and increases the risk of mortality and morbidity from inflammatory, infectious and neoplastic diseases. Short-duration breastfeeding or exclusive formula feeding reduces transmission. In Peru, where breastfeeding is common and populations affected by HTLV-1 lack access to safe water and sewage, which hinders control, understanding women’s knowledge, attitudes, and practices regarding HTLV-1 mother-to-child transmission (MTCT) is essential. We conducted a descriptive, exploratory cross-sectional survey at the Instituto de Medicina Tropical Alexander von Humboldt (Lima, Peru) between September and December 2022. Women aged 18–60 years living with HTLV-1 completed a 30-item instrument (29 closed-ended, one open-ended) assessing knowledge of HTLV-1 MTCT, attitudes toward pregnancy and breastfeeding, and related practices. Descriptive statistics were calculated, and free-text responses were thematically summarized. Of 45 survey entries, 43 unique responses were analyzed (median age 45 years [IQR 38-55.5]). Most participants resided in Metropolitan Lima (77%) and had access to water and sewage services (98%), limiting generalizability to higher-burden settings in Peru. Knowledge of HTLV-1 transmission was high: sexual transmission (95%), breastfeeding (93%), and blood transfusion (86%). Among women with children (N = 40), only nine had been diagnosed prior to any pregnancy; six recalled receiving breastfeeding counseling and adhered to recommendations (five exclusive formula, one breastfeeding <6 months). Three women who formula-fed (3/5, 60%) reported negative comments from relatives or friends. Thematic analysis (N = 37) identified six domains, including health-system limitations and stigma. Among care-engaged women in urban settings, knowledge and attitudes supportive of MTCT prevention were generally favorable; however, postpartum diagnosis emerged as the principal barrier for prevention. Targeted prenatal screening in high-risk groups, timely counseling, reliable access to breast-milk substitutes with safe water, provider training, and stigma mitigation strategies require evaluation in underserved and endemic settings.

## Introduction

Human T-cell Lymphotropic Virus type 1 (HTLV-1) is a chronic infectious disease with no available therapy, endemic in certain closed or isolated populations worldwide, particularly in low-income settings [1]. People living with HTLV-1 are exposed to higher all-cause mortality (RR 1.57; 95% CI 1.37-1.80), especially when coinfected with Human Immunodeficiency Virus (HIV), *Mycobacterium tuberculosis*, *M. leprae*, or Hepatitis C virus [1–6]. People living with HTLV-1 also experience worse quality of life related to chronic pain and inflammatory, infectious and neoplastic diseases [7,8]. HTLV-1-related diseases also aggregate in familial clusters, which may worsen household economic conditions [9].

HTLV-1 is transmitted via breastfeeding (up to 27% risk of transmission), sexual contact, and blood or organ donation [8]. In Nagasaki, Japan, Hino et al. (2011) found exclusive formula feeding significantly reduces HTLV-1 mother-to-child transmissions (MTCT) from 20.3% to 2.5% (p < 0.001), compared to breastfeeding over six months [10]. Miyazawa et al. (2021) found risk of breastfeeding for less than three months is comparable to exclusive formula feeding (RR 0.72; 95% CI 0.30-1.77), while breastfeeding up to six months almost triplicates HTLV-1 MTCT risk (RR 2.91; 95% CI 1.69-5.03) [11].

In 2011, Japan implemented a nationwide HTLV-1 MTCT prevention programme consisting of detecting pregnant women living with HTLV-1 and recommending the following: exclusive formula feeding, breastfeeding for up to three months, or frozen-thawed breast milk [12]. However, the applicability of these recommendations in low-income settings remains uncertain, because they may result in higher infant morbidity and mortality due to infectious diseases or malnutrition [13,14]. Hence, HTLV-1 MTCT prevention strategies must balance feasibility, affordability, and safety, as seen in HIV MTCT prevention efforts: early diagnosis availability, health system readiness, and culturally acceptable infant feeding alternatives are required [15,16]. Acceptability is a key outcome, as breastfeeding carries strong socio-cultural meanings [17,18].

In Central, South America and the Caribbean, the prevalence of HTLV-1 among pregnant women (1.02%; 95% CI: 0.75-1.33) is higher than that of HIV, Syphilis or Hepatitis B [19]. In middle-income settings, prenatal screening followed by breastfeeding modifications for preventing HTLV-1 MTCT is highly cost-effective when prevalence is high [20]. However, in low-income settings, short-term breastfeeding is associated with higher infant morbidity and mortality, even when breast milk substitutes and hygiene counseling are provided [21].

In Peru, breastfeeding is highly popular (up to 80% of women breastfeed for six months), and HTLV-1 mainly affects remote and culturally diverse communities with poor water and sanitation services, which interferes with infection control [22–24]. Therefore, it is necessary to understand women's perspectives around potential exposures regarding modifications in breastfeeding practices [25]. To inform the design of control strategies and guide future research, we aimed to explore the knowledge, attitudes and practices related to the HTLV-1 MTCT route among women living with the infection.

## Methods

This is a descriptive, exploratory and hypothesis-generating cross-sectional study. Data were collected at Instituto de Medicina Tropical Alexander von Humboldt (IMTAVH) in Lima, Peru, from 1 September 2022–31 December 2022. The HTLV Unit of the IMTAVH has functioned since 1989 as a reference center for specialized care for patients from across the country [23]. The attending physicians were FMC and EG.

Women living with HTLV-1 were defined as having at least two reactive serological tests, a confirmatory Western blot or proviral load measurements. We included women between 18–60 years old from the IMTAVH cohort of people living with HTLV-1 [26]. The upper age limit was chosen to reflect the age distribution of women living with HTLV-1 in care while mitigating potential recall-bias, considering the cohort includes women over 80 years old. This approach supported the exploratory aim of capturing diverse perspectives on knowledge, attitudes, and practices without restricting participation to women of reproductive age. We excluded women unable to participate due to a cognitive deficit or neurological sequelae. Sampling was non-probabilistic and based on convenience. Recruitment occurred twice per week over a four-month period in the outpatient clinic.

We designed a survey of 29 closed-ended and one open-ended question. First, we developed a draft based on three predefined domains: knowledge, attitudes and practices related to the HTLV-1 MTCT route in women living with the infection. We then added questions adapted from previous studies, including information about prenatal diagnosis and related commorbidities [27,28]. Subsequently, the instrument was revised in terms of content validity and clarity by HTLV experts (two infectious diseases specialists, two neonatologists, one epidemiologist); however, no systematic evaluation was done. Finally, FG, AP and PC piloted the survey among them and improved the wording and flow before data collection. Questions did not include clinical terminology to promote participants’ understanding. The survey lasted less than 20 minutes and is available in Spanish upon request.

The cohort’s assisting nurses informed women who fulfilled inclusion criteria about the study. We enrolled participants in the waiting room before their medical consultation. The survey was self-administered on an electronic tablet, with researchers available to provide clarification if requested; no participant requested clarification. Afterwards, participants received an informative brochure regarding the HTLV-1 MTCT. We used the cohort medical records to complement surveys with data on geographic origin and reason for diagnosis.

We collected the following baseline characteristics: age, geographic origin, residence, educational level, number of children, basic service access (electricity, water and sewage), time since HTLV-1 diagnosis, HTLV-1-related comorbidities (Tropical Spastic Paraparesis, Adult T-Cell Lymphoma/Leukemia, or Strongyloidiasis) in the participant or in a family member, and reason for HTLV-1 diagnosis. The primary outcomes were knowledge of HTLV-1 (transmission routes, breastfeeding recommendations, causal agent identification, cure availability, and related comorbidities), attitudes towards breastfeeding and future parity, and breastfeeding practices for women whose HTLV-1 diagnosis preceded pregnancies. Other variables of interest were prenatal diagnosis, breastfeeding counseling, and stigma. A secondary outcome was relevant topics for the participants, evaluated through a voluntary open-ended question.

The sample size was based on convenience. Considering the number of patients attending daily consultations and the study duration, we estimated a sample of 50 women. Given the exploratory and hypothesis-generating nature of the study, the sample size was not calculated to support inferential analyses or statistical hypothesis testing. We described categorical variables by frequencies and percentages. Qualitative data were analyzed thematically using an inductive approach, with codes generated directly from the data and without the use of a predefined analytical framework. FG and JM independently reviewed and coded responses, assigning codes to segments of text that reflected distinct ideas or concepts, then identifying recurring ideas and grouping conceptually similar codes into broader categories, from which preliminary themes and subthemes were developed. JM finally compared coding decisions, reviewed discrepancies, and refined and synthesized themes and subthemes into the final thematic structure, which was reviewed and approved by all co-authors. The anonymized database is available as a Supporting Information file. We managed data in Google Forms, Google Sheets and Stata 18.

### Ethics statement

The Institutional Research Ethics Committee (Comité Institucional de Ética en Investigación in Spanish) from Universidad Peruana Cayetano Heredia approved the study under approval number 392-34-22 and project code 208094 in August 2022. Participants provided a written informed consent after understanding the project’s objective and activities. We obtained two copies of the informed consent form, which contained identifying information; one was retained by the participants and the other securely stored in a locked room at IMTAVH. The study did not expose participants to any major risk.

We conducted a non-systematic search of PubMed and LILACS (1997–2025) using MeSH terms “Human T-lymphotropic virus 1”, “Infectious Disease Transmission, Vertical”, and “Breast Feeding”.

## Results

We recorded 45 survey completions; two were duplicate entries. After removing duplicates, the sample comprised 43 unique women (all approached participated). The median age was 45 years (IQR 38-55.5). Table 1 summarizes baseline characteristics. About 70% of women resided in Metropolitan Lima and completed secondary or tertiary education. Only 7% had no children and just 2% lacked access to basic services. Moreover, almost 50% of participants indicated at least one comorbidity related to HTLV-1.

**Table 1:** Baseline characteristics of women living with HTLV-1 attending the Instituto de Medicina Tropical Alexander von Humboldt, Lima, Peru, 2022 (N = 43).

FEATURE	DETAIL (N = 43)
**Age**	45 (IQR 38-55.5)
**Geographic origin**	
Andes	22 (51%; 95% CI ^a^: 37%-65%)
Lima	11 (26%; 95% CI: 15%-40%)
Amazon	7 (16%; 95% CI: 8%-30%)
Coast	1 (2%; 95% CI: 0%-12%)
Unknown	2 (5%; 95% CI: 1%-15%)
**Residence**	
Metropolitan Lima	33 (77%; 95% CI: 62%-87%)
Lima province	2 (5%; 95% CI: 1%-15%)
Other provinces	8 (19%; 95% CI: 10%-33%)
**Educational level**	
No education	1 (2%; 95% CI: 0%-12%)
Primary	12 (28%; 95% CI: 17%-43%)
Secondary	18 (42%; 95% CI: 28%-57%)
Tertiary	12 (28%; 95% CI: 17%-43%)
**Number of children**	
0	3 (7%; 95% CI: 2%-19%)
1	11 (26%; 95% CI: 15%-40%)
2 or more	29 (67%; 95% CI: 53%-80%)
**Basic services access**	
Electricity	43 (100%; 95% CI: 92%-100%)
Water and sewage	42 (98%; 95% CI: 88%-100%)
**Time since HTLV-1 diagnosis**	
<1 year	11 (26%; 95% CI: 15%-40%)
1-5 years	17 (40%; 95% CI: 26%-54%)
>5 years	15 (35%; 95% CI: 22%-50%)
**HTLV-1-related comorbidities** ^**b**^	
In participant	20 (47%; 95% CI: 33%-61%)
In family member	14 (33%; 95% CI: 20%-47%)
**Reason for HTLV-1 diagnosis**	
Related comorbidity	18 (42%; 95% CI: 28%-57%)
Family cluster	10 (23%; 95% CI: 13%-38%)
Research	6 (14%; 95% CI: 7%-27%)
Blood bank screening	6 (14%; 95% CI: 7%-27%)
Unknown	3 (7%; 95% CI: 2%-19%)

^a^Confidence intervals.

^b^Tropical Spastic Paraparesis, Adult T-Cell Lymphoma/Leukemia or Strongyloidiasis.

Regarding knowledge of HTLV-1 (Table 2), more than 90% of women identified sexual intercourse and breastfeeding as routes of transmission, and almost 80% correctly identified not breastfeeding or breastfeeding for up to three months as MTCT prevention strategies. When stratified by time since HTLV-1 diagnosis, differences in knowledge patterns were observed. Women diagnosed within the past year (median age 48 years [IQR 36–58]) identified fewer transmission routes and breastfeeding recommendations. Women diagnosed 1–5 years earlier (median age 48 years [IQR 40.5-56.5]) identified more frequently the causal agent, the lack of curative treatment, and HTLV-1-related comorbidities. Women diagnosed more than five years earlier (median age 44 years [IQR 33–54]) selected a higher number of incorrect transmission routes.

**Table 2:** Knowledge of HTLV-1 among women living with HTLV-1 attending the Instituto de Medicina Tropical Alexander von Humboldt, Lima, Peru, 2022 (N = 43).

FEATURE	TIME SINCE HTLV-1 DIAGNOSIS			TOTAL (N = 43)
	<1 YEAR (N = 11)	1-5 YEARS (N = 17)	>5 YEARS (N = 15)	
**Transmission routes**				
Sexual intercourse	9 (82%)	17 (100%)	15 (100%)	41 (95%; 95% CI ^a^: 85%-99%)
Breastfeeding	9 (82%)	16 (94%)	15 (100%)	40 (93%; 95% CI: 81%-98%)
Blood transfusion	7 (64%)	16 (94%)	14 (93%)	37 (86%; 95% CI: 73%-93%)
**Incorrect transmission routes**				
Contaminated toilet seat	2 (18%)	1 (6%)	3 (20%)	6 (14%; 95% CI: 7%-27%)
Mosquito bite	1 (9%)	2 (12%)	2 (13%)	5 (12%; 95% CI: 5%-24%)
Hugging	0	1 (6%)	0	1 (2%; 95% CI: 0%-12%)
**Breastfeeding recommendations**				
Know	7 (64%)	15 (88%)	12 (80%)	34 (79%; 95% CI: 65%-89%)
Don't know	4 (36%)	2 (12%)	3 (20%)	9 (21%; 95% CI: 11%-35%)
**Causal agent identification**				
Virus	8 (73%)	16 (94%)	13 (87%)	37 (86%; 95% CI: 73%-93%)
Other (Bacteria, Parasite, Fungus)	3 (27%)	1 (6%)	2 (13%)	6 (14%; 95% CI: 7%-27%)
**Cure availability**				
Not available	6 (55%)	16 (94%)	14 (93%)	36 (84%; 95% CI: 70%-92%)
Available	1 (9%)	0	0	1 (2%; 95% CI: 0%-12%)
Don’t know	4 (36%)	1 (6%)	1 (7%)	6 (14%; 95% CI: 7%-27%)
**Related comorbidities**				
Tropical Spastic Paraparesis	9 (82%)	16 (94%)	14 (93%)	39 (91%; 95% CI: 78%-96%)
Adult T-cell Lymphoma/Leukemia	7 (64%)	14 (82%)	8 (53%)	29 (67%; 95% CI: 53%-80%)
Strongyloidiasis	4 (36%)	7 (41%)	7 (47%)	18 (42%; 95% CI: 28%-57%)

^a^Confidence intervals.

Regarding attitudes and practices among 22 (51%) women aged 18–45 years: 14 did not desire a future pregnancy due to reasons different than the HTLV-1 infection, four did not desire a future pregnancy due to the HTLV-1 infection, one was unsure, and three desired another pregnancy. Among these three women with gestational desire, two would not breastfeed and the other would breastfeed for less than six months. Among 21 (49%) women aged 46–60 years: 17 did not desire a future pregnancy due to reasons different than the HTLV-1 infection, while four did not desire a future pregnancy due to the HTLV-1 infection.

Among 40 women with children, nine had been diagnosed with HTLV-1 prior to any pregnancy. Among these nine women with a prenatal diagnosis, six recalled receiving breastfeeding counseling and followed the recommendations. Among these six women, five opted for exclusive formula feeding and one for breastfeeding for less than six months. Among the five women who chose formula feeding, three reported negative comments from friends or relatives about this practice.

Thirty seven (86%) participants responded to the open-ended question (Table 3). Themes were not mutually exclusive. Six main themes emerged: (1) information, education and communication; (2) detection, prevention, and control; (3) research, treatment, and cure; (4) emotional experience and coping; (5) health system limitations; and (6) social dimension and stigma. Participants emphasized a lack of information and awareness about HTLV-1 among both the general public and healthcare providers. They called for wider education campaigns and routine HTLV-1 screening during pregnancy to prevent MTCT. Other recurring themes included the need for further research and treatment options, as well as the emotional impact of a diagnosis, which was described as shocking or distressing.

**Table 3:** Relevant HTLV-1 topics identified by women living with HTLV-1 attending in Instituto de Medicina Tropical Alexander von Humboldt, Lima, Peru, 2022 (N = 37).

THEME	SUBTHEME	TRANSLATED CITATION
**Information, education and communication**	Need for public awareness	“More people should know about this virus to prevent infections.”
	Confusion with HIV ^a^	“People think it’s the same as HIV, but it’s not.”
	Inconsistent medical information	“Some doctors tell me one thing, others another. I’m confused.”
**Detection, prevention and control**	Screening and maternal testing	“Every pregnant woman should be tested, and the test should be free.”
	Prevention of MTCT ^b^	“Women with HTLV-1 should avoid breastfeeding to protect their children.”
**Research, treatment, and cure**	Demand for research	“They should study this virus more to find a cure.”
	Treatment limitations	“There’s no Baclofen now. I wish there was an alternative.”
**Emotional experience and coping**	Psychological impact	“When I was told, it was like hearing I had cancer. I wanted to die.”
	Resilience and faith	“Now I try to teach others. It helps me too.”
**Health system limitations**	Lack of specialists and decentralization	“In the provinces there’s infrastructure but no trained personnel.”
**Social dimension and stigma**	Stigma and misinformation	“The first step against stigma is information.”
	Sensitive communication	“They should inform us without causing unnecessary fear.”

^a^Human Immunodeficiency Virus.

^b^Mother-to-child transmission.

## Discussion

This study identifies the lack of prenatal diagnosis as a key structural barrier to HTLV-1 MTCT prevention among care-engaged women living with HTLV-1 in Peru. Despite generally favorable knowledge and attitudes, most diagnoses occurred postpartum (31/40), limiting opportunities for timely counseling, breastfeeding modifications, and MTCT prevention. However, our sample reflects middle- or high-income women who receive specialized care and have access to basic services that permit adherence to breastfeeding recommendations. Therefore, our results cannot be generalized to women from communities with the highest HTLV-1 burden in the country. This study informs future research and policy, mainly on the need to understand and address the operational and ethical complexities of HTLV-1 MTCT in underserved settings.

This study underscores a critical gap between individual-level preparedness and system-level capacity and opportunity for prevention. Women correctly identified breastfeeding as a MTCT route, and recognized and showed favorable attitudes towards preventive measures, likely reflecting participants’ engagement in high-quality care and their generally high socioeconomic conditions [29]. Nevertheless, even this best-case scenario (characterized by high knowledge, favorable attitudes, and nearly universal access to basic services) was insufficient to prevent HTLV-1 MTCT, as most participants received a delayed diagnosis. However, establishing HTLV-1 prenatal diagnosis in Peru is not straightforward and it does not guarantee MTCT prevention, especially because HTLV-1 affects primarily rural, indigenous, and resource-limited communities from the Amazon and the Andes, where women face additional structural constraints and breastfeeding is a common practice [19,22,24].

In the current Peruvian context, a risk-based prenatal HTLV-1 screening could be feasible and cost-effective in the near-term [19,20]. Women from high-prevalence settings (family history of infection, sex-workers, endemic communities such as Shipibo-Konibo, and Amazon and Andes regions) could benefit from a prenatal HTLV-1 screening; however, the implementation of such intervention remains highly complex and must consider each local context [9,19,24]. Essential operational prerequisites are adequate counseling capacity (including trained interdisciplinary care providers), uninterrupted access to safe water and sustained provision of breast-milk substitutes [12,30]. Adopting a prenatal HTLV-1 screening without designing strategies to address these structural challenges would not be appropriate due to the higher infant morbidity and mortality risk [13,14]. Additionally, ethical pressures around culturally appropriate counseling and stigma related to modifying breastfeeding practices should be addressed before designing a HTLV-1 MTCT prevention program in order to increase its early acceptability and future sustainment [12,13,18,31]. In the long-term, universal prenatal screening may become cost-effective and appropriate, provided that operational, ethical, and cultural constraints are adequately addressed beforehand, and was viewed as acceptable by some participants [32,33]. Furthermore, if effective therapies to reduce HTLV-1 MTCT become available, universal prenatal screening could also permit breastfeeding recommendations different from restriction or contraindication, similar to what happened with recommendations to prevent HIV MTCT [34].

Comparisons with other endemic and resource-limited settings highlight how system-level support, rather than knowledge alone, shapes adherence to breastfeeding recommendations. Adherence to breastfeeding recommendations is a complex phenomenon that is associated with socioeconomic conditions, access to care, health literacy, counseling quality, and healthcare providers’ knowledge, themes also highlighted by our participants [30,35]. In French Guiana, prenatal HTLV-1 screening has been in place since 1995, accompanied by recommendations to avoid breastfeeding [27]. Oni et al. (2006) studied 29 women living with HTLV-1 in resource-limited settings and found that, despite limited knowledge and lack of support with breast milk substitutes, 72% of those with a prenatal diagnosis followed the advice to not breastfeed [27]. In Jamaica, Cooper et al. (2010) evaluated 88 women, including 21 living with HTLV-1, in resource-limited settings, five years after counseling to breastfeed for up to 6 months, followed by the provision of breast-milk substitutes for months 6–12 to reduce HTLV-1 MTCT [28]. Unlike our sample, fewer than half of women in that study knew their HTLV-1 serostatus, reflecting structural gaps, and whether participants adhered to the breastfeeding recommendations was not clearly reported [28]. Regardless of the low socioeconomic status and lack of current engagement in care of participants, the authors reported knowledge patterns similar to ours regarding HTLV-1 MTCT and the preventive role of breastfeeding modifications, possibly due to the availability of breast-milk substitutes in that context [36,37]. By contrast, participants in our study showed greater awareness of sexual transmission routes and HTLV-1-related diseases, likely reflecting differences in access to care [28].

Although this study was not designed to assess differences between subgroups, we observed variation in knowledge according to time since HTLV-1 diagnosis. This pattern may reflect differences in exposure to medical counseling over time, as women with more recent diagnoses may have had fewer counseling encounters, while those diagnosed many years earlier may experience partial recall of previously received information. These differences appear unrelated to age. These observations should be interpreted as hypothesis-generating and warrant further investigation in larger, longitudinal studies.

This study has several limitations. First, the generalizability of our findings is restricted by selection bias. Participants were urban, care-engaged women attending a referral center in Lima, many of whom could afford substantial out-of-pocket costs for high-quality care. As such, the findings likely represent a privileged scenario and may overestimate knowledge, acceptability, and adherence to HTLV-1 MTCT prevention strategies compared with women in socioeconomically marginalized settings. Second, the sample size was small (N = 43) and based on convenience sampling. While appropriate for a descriptive, exploratory study, this limits inference and impedes causal interpretation. Differences observed across subgroups should therefore be interpreted cautiously and viewed as descriptive patterns rather than definitive associations. Third, the qualitative component was limited to a single open-ended question. Although this question provided valuable insights into women’s experiences, particularly regarding stigma, emotional impact, and interactions with the health system, the qualitative findings represent surface-level insights rather than an in-depth exploration of these themes. More comprehensive qualitative or mixed-methods approaches would be needed to fully understand decision-making processes, social dynamics, and lived experiences related to HTLV-1 diagnosis and MTCT prevention. Fourth, the survey instrument did not undergo systematic procedures to ensure its content validity and clarity, was not piloted among a sample of participants before its execution, and may have limited ability to discriminate between varying levels of knowledge in a highly informed, care-engaged population. These limitations underscore the need for studies that integrate broader sampling strategies with deeper qualitative inquiry and a more rigorous quantitative component.

Despite these limitations, the study provides important evidence that individual knowledge and willingness alone are insufficient to prevent HTLV-1 MTCT in the absence of timely diagnosis and supportive health system conditions. Future research should prioritize implementation studies evaluating feasible and ethical strategies for delivering HTLV-1 MTCT prevention in endemic settings, accounting for health system readiness, counseling capacity, access to safe water and breast-milk substitutes, and stigma mitigation. In parallel, qualitative and mixed-methods studies are needed to deepen understanding of stigma, emotional burden, and decision-making processes. This should include perspectives of male partners in gender-normative contexts and the potential role of women living with HTLV-1 as peers or advocates [25]. Further work should also examine how these findings differ in rural, Amazonian and Andean contexts, where structural constraints and social dynamics may substantially alter prevention feasibility. Finally, longer-term research priorities include the development of effective therapies for people living with HTLV-1.

## Conclusions

Among urban, care-engaged women living with HTLV-1 in Peru, knowledge and attitudes supportive of MTCT prevention were generally high. However, individual knowledge and willingness alone are insufficient in the absence of prenatal diagnosis and supportive health system conditions. Although this study represents a best-case scenario and cannot be generalized to higher-burden or underserved settings, it highlights the need for context-specific, ethically grounded strategies to improve HTLV-1 MTCT prevention. Future efforts should focus on implementation research to evaluate feasible screening approaches, strengthen counseling capacity, and ensure equitable access to the resources required to support prevention in endemic settings.

## Supporting information

S1 Data Anonymized database of 43 women living with HTLV-1 attending the Instituto de Medicina Tropical Alexander von Humboldt, Lima, Peru, 2022. (XLSX)
