# Supplementary material for: Women’s knowledge, attitudes and practices related to mother-to-child transmission of HTLV-1 in Peru
Source: PLOS Glob Public Health. 2026 Aug 27;6(8):e0007103. doi: 10.1371/journal.pgph.0007103 (PMC13521394; doi:10.1371/journal.pgph.0007103)
Supplement: Supplementary file 1 [file pgph.0007103.s005.pdf]

Review date: 30 JUNE 2026

Comments or suggestions for authors

Abstract

1. The suggested title would be "Women's knowledge, attitude and practices related to transmission of Human T-Cell Lymphotropic Virus Type-1 through breastfeeding in Peru". [Explanation: Better to avoid any short forms in the title; To make the title easy and readers understandable, only knowledge assessment can give insights about attitude and practice. However, authors can subsequently discuss about attitude and practice based on assessing the knowledge]
2. Can it cause leukemia or other diseases? If so, please start with…" …can be transmitted from mother to child through breastfeeding which can cause leukemia or other serious diseases". [Explanation: Readers are interested to know the effect of virus]
3. Lack access to safe water is not direct cause of transmission. Hence, it is recommended to omit such words in the abstract, if irrelevant.
4. Recommended to avoid "…many population". [Explanation: "many" is a vague term, not suitable in the abstract].
5. Small sample size and inadequate statistics. Please, mention some basic socio-demographic status of the respondents such as education [e.g. % of respondents were post-graduate/graduate/completed primary school education], occupation [e.g. % of respondents had jobs, business, housewife and others], marital status etc.
6. Please explain why water and swage services are associated with transmission of HTLV-1 as it the HTLV-1 transmit through blood, breastmilk and sexual intercourse.
7. The higher rates [e.g. more than 85%] of knowledge on three core routes of transmission is surprising for such rare virus transmission. Please check and justify.
8. Please capture the source of knowledge and rates of knowledge sources [e.g. breastfeeding counseling, media advertisement/health education/billboard/mobile SMS/social media]
9. Please clearly state and rate the practice related stigma [e.g. formula-feed]. It is recommended to describe the rate instead of number of respondents.

Introduction

1. Line number 50, please avoid insertion of RR, CI with references. It is recommended to insert all references at the end of the sentence. Authors are suggested to review more articles to shape citation style.
2. Line number 57-58, recommended to insert reference [10] at the end of the sentence. Similarly, for other sentences. In addition, recommended to use recent citation instead of old references.
3. From line number 63, recommended to start explaining about knowledge, attitude and practice related to HTLV-1 transmission.
4. The line number from 64 to 73, authors nicely mentioned the interventions in Japan, its feasibility in low-income settings and emphasis on strategic balance of interventions based on socio-demographic and socio-cultural factors. Please also discuss about knowledge and practice related to maternal and child transmission of HTLV-1. Please explain what level of

knowledge the researchers found in different settings [e.g. % mothers had higher and ---% of mother had average knowledge, cut-off point for higher, average and below level knowledge related to MTCT, also discuss which occupation group or income groups had higher knowledge, urban-rural difference – if any].

5. The line number 75-76, authors mentioned higher prevalence of HTLV-1 than other major diseases. It is recommended to insert the percentage of each disease. In line 76, please revise the sentence as past form [e.g. “was” instead of “is”].
6. In line number 76-78, prenatal screening is cost-effective to prevent transmission of HTLV-1; Please also discuss about knowledge sources [e.g. screening, social media and or mass media advertisement, community sessions].

Methods

1. In the line 95, please omit the names of doctors or clinicians to ensure anonymization. According to Helsinki declaration, it is necessary to avoid any names. You can acknowledge their contribution in acknowledgement section.
2. In the line number 99, elaborate the short form of IMTAVH and the line number 97-98 can be inserted after the line number 99. In line 98, please mention the number of participants you have included. The age, occupation, income, residence, history of exposure to HTLV-1, co-morbidity, inclusion and exclusion criteria and other information required for this study.
3. In the line number 114, elaborate the short forms of FG, AP and PC.
4. The sentence from 120-122, need improvement to make it understandable.
5. The line number 126-130, information can be replaced with above mentioned area.
6. In the line 150, authors estimated 50 samples. Please clearly state the actual number of enrolled participants.
7. In the line number 151, authors mentioned funding information. It is recommended to exclude funding information in methods section. It is necessary to mention separately.
8. Please mention the scientific steps of participant’s enrollment or a sub-heading of consent to participate such as sharing of study objectives, anonymization, risks and benefits, tentative time of interview, methods of interview (e.g. face to face or other process), freedom of acceptance or rejection and other points of Helsinki declaration. Then mention whether you received a written informed consent.

Results

1. In table 1., Huge correction required. The baseline information should be age [e.g. age range – 18-45year, 46-60 year], education, occupation, income, residence, religion, smoking and diabetes status, and other socio-demographic information [e.g. marital status]. CI range and 95% of CI wrongly presented which is not essential for table 1. In addition, for the value 1, the CI range 0-12 is usually not acceptable.
2. Line number 182 to 187, need proper clarification.

3. For table 2., authors are requested to learn more about organization of tables utilizing statistical software [e.g. stata], capturing the key facts, how to write CI range.
4. Authors are suggested to group the age range. Was it only two age groups?

Discussion

1. In the line 228, please state how you declared middle- or high-income women.
2. There is scope of improving English throughout the discussion and other parts of the manuscript.

References

1. Recommended to replace old references with recent references.

Overall: The study was highly technical and not properly presented/organized. The statistical analysis and organization of tables were not adequately inserted with lack of interpretation.
