# Supplementary material for: Women’s knowledge, attitudes and practices related to mother-to-child transmission of HTLV-1 in Peru
Source: PLOS Glob Public Health. 2026 Aug 27;6(8):e0007103. doi: 10.1371/journal.pgph.0007103 (PMC13521394; doi:10.1371/journal.pgph.0007103)
Supplement: Supplementary file 2 [file pgph.0007103.s003.docx]

PLOS Global Public Health

PGPH-D-25-03721

Women’s knowledge, attitudes and practices related to mother-to-child transmission of HTLV-1 in Peru

Dear Editor and Reviewers,

We sincerely thank the Editor and Reviewers for their thoughtful and constructive feedback. We have carefully addressed all comments and revised the manuscript accordingly. Below, we provide a concise point-by-point response. When comments overlapped conceptually, we addressed them through integrated revisions, which we note explicitly.

Editor’s comments

Comment: Strengthen emphasis on external validity, clarify rigor of the qualitative component, and frame findings as exploratory evidence to inform ethically grounded screening strategies.

Response: We have substantially revised the Abstract, Methods, Discussion, Limitations, and Conclusions to emphasize that this study represents a best-case scenario among urban, care-engaged women in Lima, and that findings are not generalizable to higher-burden or underserved settings. We reposition postpartum diagnosis as the central structural barrier to MTCT prevention and explicitly frame the study as descriptive, exploratory, and hypothesis-generating. We also expanded discussion of ethical and operational prerequisites for both risk-based and universal screening strategies, including counseling capacity, access to safe water and breast-milk substitutes, and stigma mitigation.

Reviewer #1 and Reviewer #2

Representativeness and external validity

Comment: The urban, care-engaged sample limits generalizability and may overestimate knowledge and feasibility of prevention.

Response: We agree and now explicitly frame the study population throughout the manuscript as urban women engaged in specialized care. The Abstract, Discussion, and Conclusions emphasize limited external validity and clearly state that findings likely overestimate knowledge and adherence relative to rural, Amazonian, and Andean settings. Selection bias is discussed in detail as a key limitation, and a best-case scenario framing is adopted to avoid overgeneralization.

Timing of diagnosis as a structural barrier

Comment: Postpartum diagnosis should be more prominently positioned as the central barrier to MTCT prevention.

Response: We fully agree and have repositioned postpartum diagnosis as the principal structural barrier in the Abstract, Results, Discussion, and Conclusions. We clearly state that delayed diagnosis, rather than lack of willingness or adherence, is the main obstacle to effective MTCT prevention in this context.

Qualitative component: depth and rigor

Comment: Clarify that the qualitative findings are exploratory and provide more methodological detail.

Response: We expanded the Methods section to describe how themes were identified and synthesized. We explicitly acknowledge that the qualitative component is based on a single open-ended question and provides surface-level, exploratory insights. The Limitations and Discussion now clearly state the need for more in-depth qualitative or mixed-methods research.

Language and style

Comment: Use terminology consistently and reduce repetition

Response: We ensured consistent terminology throughout the manuscript, reduced repetition in the Discussion, and improved overall flow and clarity.

Reviewer #1

High knowledge levels and counseling exposure

Comment: High knowledge likely reflects repeated counseling rather than baseline awareness.

Response: We revised the Discussion to explicitly interpret high knowledge levels as reflecting prolonged engagement with specialized care and repeated counseling, and to clarify that findings do not reflect community-level knowledge.

Screening strategies and ethical considerations

Comment: Clarify distinctions between risk-based and universal screening and acknowledge ethical tensions around breastfeeding.

Response: We revised the Discussion to clearly distinguish between risk-based and universal antenatal screening approaches and to emphasize ethical and operational prerequisites, including counseling capacity, safe water access, availability of breast-milk substitutes, and stigma mitigation. Ethical tensions related to breastfeeding norms and infant risk are now explicitly addressed.

Reviewer #2

Sample size, analytic scope, and exploratory nature

Comment: Small convenience sample and descriptive analyses limit inference.

Response: We explicitly state in the Methods and Limitations that the study is descriptive, exploratory, and hypothesis-generating. We justify the sample size and explain why inferential analyses were not pursued. Stratified descriptive analyses (by age group and time since diagnosis) were added to enhance analytic depth without overinterpretation.

Ceiling effect and instrument limitations

Comment: High knowledge levels raise concern for ceiling effects and social desirability bias.

Response: We now discuss the potential ceiling effect of the knowledge instrument and acknowledge its limited ability to discriminate between varying levels of knowledge in a highly informed, care-engaged population. This limitation is explicitly addressed in the Discussion and Limitations.

System-level barriers to antenatal diagnosis

Comment: Expand discussion of health-system barriers and link findings to screening feasibility.

Response: We expanded the Discussion to address system-level barriers to antenatal HTLV-1 diagnosis, including lack of screening policies and provider awareness. We strengthened the link between postpartum diagnosis and the need for context-specific screening strategies, framed within an implementation research perspective.

Tables, results presentation, and clarity

Comment: Complete table titles, clarify denominators, and improve readability.

Response: All table titles now include population, location, study period, and sample size. Denominators are clarified consistently across subgroups. Incorrect transmission routes were grouped to improve readability, minor numerical inconsistencies were corrected, and knowledge results were stratified by time since diagnosis to improve interpretability.

Comparison with HIV prevention strategies

Comment: Frame carefully and avoid direct equivalence

Response: Comparisons with HIV prevention strategies and with other settings were revised to emphasize system-level barriers and contextual differences rather than direct equivalence.

We are grateful for the reviewers’ insightful feedback, which has substantially strengthened the clarity, rigor, and policy relevance of this manuscript. We hope the revisions adequately address all concerns and look forward to your assessment.

Kind regards,

Dr. Eduardo Gotuzzo

Instituto de Medicina Tropical Alexander von Humboldt

Universidad Peruana Cayetano Heredia
