# Supplementary material for: Women’s knowledge, attitudes and practices related to mother-to-child transmission of HTLV-1 in Peru
Source: PLOS Glob Public Health. 2026 Aug 27;6(8):e0007103. doi: 10.1371/journal.pgph.0007103 (PMC13521394; doi:10.1371/journal.pgph.0007103)
Supplement: Supplementary file 3 [file pgph.0007103.s004.docx]

PLOS Global Public Health

PGPH-D-25-03721R1

Women’s knowledge, attitudes and practices related to mother-to-child transmission of HTLV-1 in Peru

Dear Editor and Reviewers,

We thank the Reviewers for their recognition and constructive feedback. We have addressed each of the comments and revised the manuscript accordingly. A concise point-by-point response is provided below.

Reviewer #2:

Comment: A small amount of repetition remains in the discussion regarding postpartum diagnosis as a barrier to prevention. Minor editorial streamlining could improve conciseness.

Response: We eliminated repetitive wording regarding “postpartum diagnosis as a barrier” and “prenatal screening” in the discussion to improve readability. We also detected and reduced redundancy in the conclusion.

Reviewer #3:

Comment: The descriptive statistical approach is appropriate. The authors may consider reporting 95% confidence intervals for key proportions to provide additional context regarding the precision of the estimates.

Response: We included 95% confidence intervals (CI) for key proportions in Tables 1 and 2 to provide additional information on the precision of estimates. CIs were calculated using the Wilson score method for binomial proportions.

Comment: Clarify the Description of the Qualitative Analysis. The qualitative component is based on a single open-ended question and appropriately complements the quantitative findings. To enhance methodological transparency, the authors may briefly describe how responses were grouped into themes and summarized. A concise explanation of how themes were identified would improve clarity without requiring extensive qualitative detail.

Response: We expanded the qualitative component to include that theme identification was through an inductive approach, consisting in identifying and grouping recurring ideas and similar responses.

Comment: Clarify Survey Development and Validation. The authors note that the questionnaire was developed by HTLV experts and piloted prior to data collection. Providing additional detail on its development (such as its basis in prior studies, pilot testing procedures, and any steps taken to ensure clarity and content validity) would strengthen the methodological rigor of the manuscript.

Response: We reorganized and presented in detail the stepwise survey development and validation. We now include a chronological development description of the survey: first draft, literature contribution, expert review, and pilot refinement.

Comment: Minor Editorial Suggestions. The limitations section appropriately acknowledges selection bias. Expanding this discussion to address implications for rural and underserved populations in Peru would further enhance the manuscript’s public health relevance. Ensure consistency in terminology throughout the manuscript. After defining ‘mother-to-child transmission (MTCT)’ at first use, the abbreviation should be used consistently in the main text to enhance clarity and readability.

Response: We already address the implications of our results for underserved (Amazonian and Andean) settings in Peru, discussing context-specific structural constraints and social dynamics (lines 241-256, 323-325). We ensured abbreviations are used consistently throughout the manuscript.

We are grateful for the reviewers’ feedback, which has strengthened the rigor of this manuscript even more. We hope the revisions address all concerns and look forward to your assessment.

Kind regards,

Dr. Eduardo Gotuzzo

Instituto de Medicina Tropical Alexander von Humboldt

Universidad Peruana Cayetano Heredia
