# Supplementary material for: Women’s knowledge, attitudes and practices related to mother-to-child transmission of HTLV-1 in Peru
Source: PLOS Glob Public Health. 2026 Aug 27;6(8):e0007103. doi: 10.1371/journal.pgph.0007103 (PMC13521394; doi:10.1371/journal.pgph.0007103)
Supplement: Supplementary file 4 [file pgph.0007103.s006.docx]

PLOS Global Public Health

PGPH-D-25-03721R2

Women’s knowledge, attitudes and practices related to mother-to-child transmission of HTLV-1 in Peru

Dear Editor and Reviewers,

We thank the Editor and Reviewers for their feedback. We have addressed the comments and revised the manuscript, as described below.

Editor comment: Additional detail regarding the qualitative analysis, the validation and pilot testing of the questionnaire and the policy implications of the generated results

Response: We have added further specifications on both the qualitative analysis and the questionnaire development and validation, as well as expanded the limitations section for more transparency. We have also elaborated on the policy implications of the study to enhance impact, including a deeper discussion of the implementation challenges for reducing the mother-to-child transmission of HTLV-1 in Peru.

Reviewer #4 comments: Several aspects require clarification or refinement to improve methodological transparency, interpretability, and overall impact.

Qualitative analysis: Describe clearer the coding processes, themes and subthemes identification, and intercoder consistency assurance.

Response: We have given a more detailed description of the entire qualitative analysis processes (lines 142-150) to increase the results interpretability.

Survey development and validation: Elaborate on questionnaire development, piloting details, and content validity and clarity.

Response: A stepwise description of the questionnaire development was already present (lines 108-117), including the role of piloting. We added detail about the role of prior studies, described that no specific procedures were used by experts to ensure content validity or clarity, and expanded the limitations section accordingly.

Policy and implementation implications: Expand on implementation challenges, operational feasibility, resource requirements, ethical and cultural considerations, and integration into existing maternal health programs of prenatal screening as a potential intervention.

Response: We extended the discussion paragraph about the current challenges, research and policy considerations for implementing prenatal screening (lines 249-267), since an Implementation Science perspective.

We are grateful for the reviewers’ feedback, which has strengthened this manuscript even more. We hope the revisions address all concerns and look forward to your assessment.

Kind regards,

Dr. Eduardo Gotuzzo

Instituto de Medicina Tropical Alexander von Humboldt

Universidad Peruana Cayetano Heredia
