# Supplementary material for: Women’s knowledge, attitudes and practices related to mother-to-child transmission of HTLV-1 in Peru
Source: PLOS Glob Public Health. 2026 Aug 27;6(8):e0007103. doi: 10.1371/journal.pgph.0007103 (PMC13521394; doi:10.1371/journal.pgph.0007103)
Supplement: Supplementary file 5 [file pgph.0007103.s007.docx]

PLOS Global Public Health

PGPH-D-25-03721R3

Women’s knowledge, attitudes and practices related to mother-to-child transmission of HTLV-1 in Peru

Dear Editor and Reviewers,

We thank the Reviewers for their feedback. We have addressed the comments, polished the manuscript redaction globally to improve readability, and also explained disagreement points below.

Reviewer #5 comments and our responses respectively:

To change the title to include HTLV-1 long form – The title describes the project nature in less than 15 words, which is better for readability. HTLV-1 meaning is present in the abstract.

To include serious complications in the abstract – We expanded on the higher mortality and morbidity related to the infection in the abstract to call the attention of readers.

To explain why water and sewage services are associated with HTLV-1 transmission in the abstract – We expanded on the connection between basic services and infection control in the abstract. This relationship is fully described in the Introduction and related to policy implications in the discussion.

To include sociodemographic information of respondents in the abstract – The abstract included most relevant sociodemographic information about women from middle- or high-income urban settings who participated.

To state rates of stigma in the abstract – We added the denominator and percentage of women who experienced stigma among those who formula-fed their infants, which better exposes the problem.

To explain about knowledge, attitudes and practices related to HTLV-1 MTCT in the introduction – A thoughtful reflection on knowledge, attitudes and practices related to HTLV-1 MTCT is present in discussion, where it is also contrasted with our results.

To insert the percentage of diseases less prevalent than HTLV-1 in the introduction – We included the prevalence of HTLV-1. The message of HTLV-1 being as important for women as other infections is now well exposed and the writing remains understandable.

To omit clinicians names and elaborate on initials in methods – We changed full names for initials. We kept initials in methods because author roles directly affect the project settings, e.g. attending physicians did not enroll participants, which could have introduced biases.

To include the number of participants and baseline characteristics on methods – We included this information in results.

To exclude funding information from methods – We excluded funding sources from methods, as it is stated elsewhere.

To expand on the informed consent steps – We elaborated on the informed consent process, focusing on participants' understanding of the project.

To modify table presentation – We revised table formats to make them suitable for publication. The available demographic data are present on tables and in prose in results. Subgroups descriptions (reproductive age and time since HTLV-1 diagnosis) permit better interpretability and understanding of results implications.

To check and justify high rates of knowledge on transmission routes of women who participated – We verified our data and there were no changes. In the discussion, we debated about the reasons why participants were high-performers (care-engaged population).

To capture the sources of knowledge – Data collection of this study finished in 2022 and we did not collect data regarding the knowledge source.

To modify references style and use recent references – We displaced references at the end of sentences as recommended. We are referring to the most appropriate reading sources to understand the problem and follow the discussion.

To improve English and replace vague terms – We have verified grammar, sentence structure and understandability. We have also replaced ambiguous or imprecise terminology.

Reviewers #3 and #4 considered we addressed all previous reviewers’ comments. Data are appropriately and rigorously described, are fully available as supporting information and directly support our conclusions.

We have rigorously addressed previous review rounds and consider our manuscript is ready for publication. We are grateful for the reviewers and editors’ feedback, which substantially improved our writing. This work is already informing future research and policy benefiting women living with HTLV-1 in underserved contexts in Peru.

Kind regards,

Dr. Eduardo Gotuzzo

Instituto de Medicina Tropical Alexander von Humboldt

Universidad Peruana Cayetano Heredia
