# Supplementary material for: Women’s knowledge, attitudes and practices related to mother-to-child transmission of HTLV-1 in Peru
Source: PLOS Glob Public Health. 2026 Aug 27;6(8):e0007103. doi: 10.1371/journal.pgph.0007103 (PMC13521394; doi:10.1371/journal.pgph.0007103)
Supplement: Peer review history

# Peer review: Decision Letter 0

**DOI:** 10.1371/journal.pgph.0007103.r001

**Authors:** Raquel Muñiz-Salazar

12 Jan 2026

PGPH-D-25-03721

Women’s knowledge, attitudes and practices related to mother-to-child transmission of HTLV-1 in Peru

PLOS Global Public Health

Dear Dr. Gotuzzo,

Thank you for submitting your manuscript to PLOS Global Public Health. After careful consideration, we feel that it has merit but does not fully meet PLOS Global Public Health’s publication criteria as it currently stands. Therefore, we invite you to submit a revised version of the manuscript that addresses the points raised during the review process.

The reviewers concur that the primary barrier to prevention is not a lack of individual motivation or information, but rather a structural deficiency within the healthcare system, which leads to diagnoses occurring predominantly in the postpartum period—thereby missing the critical window for prenatal screening. In light of this, the discussion should be strengthened with greater emphasis on the study’s external validity, a more detailed account of the rigor underpinning the qualitative analysis, and a clear presentation of the findings as an exploratory basis for future universal screening policies. These policies should be designed with careful consideration of the ethical and operational constraints specific to different regions of Peru.

**Reviewers’ decision: Major Revision.**

Please include the following items when submitting your revised manuscript:

- A letter that responds to each point raised by the editor and reviewer(s). You should upload this letter as a separate file labeled 'Response to Reviewers'.
- A marked-up copy of your manuscript that highlights changes made to the original version. You should upload this as a separate file labeled 'Revised Manuscript with Track Changes'.
- An unmarked version of your revised paper without tracked changes. You should upload this as a separate file labeled 'Manuscript'.

Guidelines for resubmitting your figure files are available below the reviewer comments at the end of this letter.

We look forward to receiving your revised manuscript.

Kind regards,

Raquel Muñiz-Salazar, Ph.D.

Academic Editor

PLOS Global Public Health

Journal Requirements:

1. For studies involving third-party data, we encourage authors to share any data specific to their analyses that they can legally distribute. PLOS recognizes, however, that authors may be using third-party data they do not have the rights to share. When third-party data cannot be publicly shared, authors must provide all information necessary for interested researchers to apply to gain access to the data. (https://journals.plos.org/plosone/s/data-availability#loc-acceptable-data-access-restrictions)

For any third-party data that the authors cannot legally distribute, they should include the following information in their Data Availability Statement upon submission:

1) A description of the data set and the third-party source

2) If applicable, verification of permission to use the data set

3) Confirmation of whether the authors received any special privileges in accessing the data that other researchers would not have

4) All necessary contact information others would need to apply to gain access to the data

If the reviewer comments include a recommendation to cite specific previously published works, please review and evaluate these publications to determine whether they are relevant and should be cited. There is no requirement to cite these works unless the editor has indicated otherwise.

Additional Editor Comments (if provided):

The reviewers concur that the primary barrier to prevention is not a lack of individual motivation or information, but rather a structural deficiency within the healthcare system, which leads to diagnoses occurring predominantly in the postpartum period—thereby missing the critical window for prenatal screening. In light of this, the discussion should be strengthened with greater emphasis on the study’s external validity, a more detailed account of the rigor underpinning the qualitative analysis, and a clear presentation of the findings as an exploratory basis for future universal screening policies. These policies should be designed with careful consideration of the ethical and operational constraints specific to different regions of Peru.

Reviewers’ decision: Major Revision.

[Note: HTML markup is below. Please do not edit.]

Reviewers' comments:

Reviewer's Responses to Questions

**Comments to the Author**

1. Does this manuscript meet PLOS Global Public Health’s publication criteria?

Reviewer #1: Partly

Reviewer #2: Yes

**********

2. Has the statistical analysis been performed appropriately and rigorously?

Reviewer #1: No

Reviewer #2: N/A

**********

3. Have the authors made all data underlying the findings in their manuscript fully available (please refer to the Data Availability Statement at the start of the manuscript PDF file)?

The PLOS Data policy

Reviewer #1: Yes

Reviewer #2: Yes

**********

4. Is the manuscript presented in an intelligible fashion and written in standard English?

Reviewer #1: Yes

Reviewer #2: Yes

**********

Reviewer #1: This manuscript addresses an important and underexplored public health issue: women’s knowledge, attitudes, and practices related to mother-to-child transmission (MTCT) of HTLV-1 in Peru. The focus on women living with HTLV-1 and their perspectives on pregnancy, breastfeeding, stigma, and health-system barriers is timely and well aligned with the scope of PLOS Neglected Tropical Diseases / PLOS Global Public Health.

The study provides original data from an HTLV-1–endemic country where prevention policies remain limited, and it highlights a critical gap between individual-level knowledge and system-level capacity for MTCT prevention. The manuscript is generally well written, ethically sound, and methodologically transparent for a descriptive cross-sectional study. With minor to moderate revisions, it would make a valuable contribution to the literature.

Major Points

1. Major Comments

A key limitation of the study is the restricted representativeness of the sample. Most participants were urban women receiving care at a national reference center in Lima, with nearly universal access to water and sanitation. While this is clearly described, the implications should be more strongly emphasized throughout the Discussion and Conclusions.

The favorable knowledge levels and reported feasibility of infant-feeding modifications likely reflect prolonged engagement with specialized care and cannot be extrapolated to rural, Amazonian, or Andean populations, where HTLV-1 burden is often higher and structural constraints are greater. The authors are encouraged to explicitly frame their findings as representative of urban, care-engaged women and to avoid overgeneralization to all women living with HTLV-1 in Peru.

The high levels of knowledge observed among participants are an important finding, but they should be interpreted in context. Knowledge likely reflects repeated exposure to counseling within a specialized reference center rather than baseline community awareness.

Importantly, the study shows that favorable knowledge and attitudes alone are insufficient for MTCT prevention when diagnosis occurs postpartum. This distinction between individual-level preparedness and structural opportunity for prevention should be made more explicit in the Discussion.

One of the most relevant findings is that most women were diagnosed after pregnancy, severely limiting opportunities for MTCT prevention. This issue should be more prominently positioned as the central structural barrier identified by the study.

The manuscript would benefit from clearly stating that delayed diagnosis, rather than lack of willingness or adherence, represents the main obstacle to effective MTCT prevention in the current context.

The inclusion of an open-ended question adds valuable insight into women’s experiences, particularly regarding stigma, emotional impact, and health-system limitations. However, the qualitative component is necessarily limited, as it is based on a single open-ended question rather than in-depth qualitative methods.

The authors should explicitly describe these findings as exploratory and hypothesis-generating, and acknowledge that further qualitative or mixed-methods research is needed to deepen understanding of these themes.

The discussion of antenatal screening is appropriate and relevant, but it would benefit from greater clarity. In particular, the manuscript should more clearly distinguish between risk-based and universal screening approaches, and briefly acknowledge the ethical and operational prerequisites for screening, such as counseling capacity, access to breast-milk substitutes, safe water, and stigma mitigation.

2. Minor Comments

The abstract could briefly acknowledge that the study population largely consisted of urban women with access to basic services, to better contextualize the findings.

Terminology related to MTCT and infant-feeding recommendations should be used consistently throughout the manuscript.

The limitations section would benefit from a slightly expanded discussion of selection bias and the limited depth of the qualitative component.

Reviewer #2: 1. General Assessment: This manuscript addresses an important and underexplored public health issue: women’s knowledge, attitudes, and practices (KAP) regarding mother-to-child transmission (MTCT) of HTLV-1 in Peru. The topic is timely and relevant, particularly given growing regional interest in HTLV-1 screening and prevention strategies. The study benefits from a clear objective, ethical rigor, and integration of quantitative and qualitative components. Overall, the manuscript is well written and coherent, and the findings provide useful insights to inform policy and future research. However, some issues related to study design, representativeness, analytic depth, and interpretation should be addressed to strengthen the manuscript’s scientific contribution and external validity.

2. Representativeness and External Validity: The study population is drawn from a specialized reference center in Lima, with most participants living in urban areas and having access to basic services. This limits generalizability, especially given that HTLV-1 in Peru disproportionately affects remote, indigenous, and resource-limited communities.

Suggestions: Explicitly emphasize in the Abstract and Discussion that findings likely represent women already engaged in care and may overestimate knowledge and adherence. Clarify how these findings might differ in endemic rural or Amazonian settings, where water access, health literacy, and stigma may be substantially different. Consider reframing conclusions to stress that results reflect “best-case” scenarios within the Peruvian context.

3. Sample Size and Analytic Scope: The sample size (n = 43) is small and based on convenience sampling, which is appropriate for an exploratory study but limits inference. Analyses are purely descriptive; no stratification or comparison is attempted.

Suggestions: Clearly state that the study is exploratory and hypothesis-generating; consider simple stratified descriptions (e.g., by age group, education level, or timing of diagnosis) to add analytic depth, even without inferential statistics; justify why inferential analyses were not pursued and why the small sample size was small.

4. Knowledge Assessment and Potential Ceiling Effect

Reported knowledge levels are very high (e.g., >90% awareness of breastfeeding transmission), which contrasts with findings from other low- and middle-income settings. This raises concern about selection bias or possible social desirability bias.

Suggestions: Discuss the possibility of a ceiling effect in the knowledge domain; clarify whether participants had received prior counseling at the HTLV unit, which could explain high knowledge levels; acknowledge that the instrument may not discriminate well between varying levels of knowledge in highly informed populations.

5. Timing of Diagnosis and Missed Prevention Opportunities

The finding that most women were diagnosed postpartum is one of the most policy-relevant results. However, this is not fully explored.

Suggestions: Expand discussion on system-level barriers to antenatal HTLV-1 diagnosis (e.g., lack of screening policies, provider awareness). Strengthen the link between this finding and recommendations for risk-based antenatal screening, clarifying feasibility in Peru.

6. Qualitative and quantitative Components: Depth and Limitations: The open-ended question provides valuable insights but is limited to a single item. Thematic analysis is briefly described, with limited methodological detail.

Suggestions: Clarify in the methods section how themes were identified (e.g., number of coders, consensus process). Explicitly acknowledge that findings represent surface-level qualitative insights, not in-depth exploration. Suggest future qualitative or mixed-methods studies to deepen understanding of stigma, emotional impact, and decision-making. Clarify whether the survey instrument is available as supplementary material or upon request. Justify why include women up to 60 years old. They are not in the reproductive period and may not recall the information. Include more data about the qualitative component. Was it interviewer-assisted?

7. Results: Complete the titles of the tables including population, location, period of the study and number of participants. In Table 2, consider grouping incorrect transmission routes (e.g., mosquito bites, toilet seats) under a single category to improve readability. Clarify denominators consistently when referring to subgroups (e.g., women diagnosed before pregnancy).

8. Discussion: The comparison with HIV prevention strategies is appropriate but should be framed carefully to avoid implying direct equivalence. Consider briefly addressing ethical tensions between breastfeeding norms and HTLV-1 prevention.

9. Language and Style: Overall clarity is strong. Minor edits could reduce repetition in the Discussion (e.g., repeated references to access to care and counseling). Ensure consistent use of terms such as “MTCT prevention,” “breastfeeding modification,” and “breast-milk substitutes.”

10. Strengths of the Study: Addresses a neglected infection with growing public health relevance. Focuses on women’s perspectives, aligning with rights-based and patient-centered approaches. Integrates qualitative insights that highlight stigma, emotional burden, and health system gaps. Provides contextually grounded recommendations relevant to Peru and similar settings.

**********

what does this mean?). If published, this will include your full peer review and any attached files.

**Do you want your identity to be public for this peer review?**  If you choose “no”, your identity will remain anonymous but your review may still be made public.

For information about this choice, including consent withdrawal, please see our Privacy Policy

Reviewer #1: **Yes:**  Tatiane Assone

Reviewer #2: No

**********

[NOTE: If reviewer comments were submitted as an attachment file, they will be attached to this email and accessible via the submission site. Please log into your account, locate the manuscript record, and check for the action link "View Attachments". If this link does not appear, there are no attachment files.]

# Author comment: Author response to Decision Letter 1

**DOI:** 10.1371/journal.pgph.0007103.r002

5 Feb 2026

Attachment Submitted filename: Response_PLOS_02_detailed.docx

# Peer review: Decision Letter 1

**DOI:** 10.1371/journal.pgph.0007103.r003

**Authors:** Helen Howard

26 Apr 2026

PGPH-D-25-03721R1

Women’s knowledge, attitudes and practices related to mother-to-child transmission of HTLV-1 in Peru

PLOS Global Public Health

Dear Dr. Gotuzzo,

Thank you for submitting your manuscript to PLOS Global Public Health. After careful consideration, we feel that it has merit but does not fully meet PLOS Global Public Health’s publication criteria as it currently stands. Therefore, we invite you to submit a revised version of the manuscript that addresses the points raised during the review process.

Please submit your revised manuscript by Jun 23 2026 11:59PM. If you will need more time than this to complete your revisions, please reply to this message or contact the journal office at globalpubhealth@plos.org. When you're ready to submit your revision, log on to https://www.editorialmanager.com/pgph/ and select the 'Submissions Needing Revision' folder to locate your manuscript file.

Please include the following items when submitting your revised manuscript:

- A letter that responds to each point raised by the editor and reviewer(s). You should upload this letter as a separate file labeled 'Response to Reviewers'.
- A marked-up copy of your manuscript that highlights changes made to the original version. You should upload this as a separate file labeled 'Revised Manuscript with Track Changes'.
- An unmarked version of your revised paper without tracked changes. You should upload this as a separate file labeled 'Manuscript'.

Guidelines for resubmitting your figure files are available below the reviewer comments at the end of this letter.

As the corresponding author, your ORCID iD is verified in the submission system and will appear in the published article. PLOS supports the use of ORCID, and we encourage all coauthors to register for an ORCID iD and use it as well. Please encourage your coauthors to verify their ORCID iD within the submission system before final acceptance, as unverified ORCID iDs will not appear in the published article. *Only*  the individual author can complete the verification step; PLOS staff *cannot*  verify ORCID iDs on behalf of authors.

We look forward to receiving your revised manuscript.

Kind regards,

Helen Howard

Staff Editor

PLOS Global Public Health

Journal Requirements:

If the reviewer comments include a recommendation to cite specific previously published works, please review and evaluate these publications to determine whether they are relevant and should be cited. There is no requirement to cite these works unless the editor has indicated otherwise.

Additional Editor Comments (if provided):

[Note: HTML markup is below. Please do not edit.]

Reviewers' comments:

Reviewer's Responses to Questions

**Comments to the Author**

Reviewer #2: All comments have been addressed

Reviewer #3: (No Response)

**********

publication criteria?

Reviewer #2: Yes

Reviewer #3: (No Response)

**********

3. Has the statistical analysis been performed appropriately and rigorously?

Reviewer #2: Yes

Reviewer #3: (No Response)

**********

4. Have the authors made all data underlying the findings in their manuscript fully available (please refer to the Data Availability Statement at the start of the manuscript PDF file)?

The PLOS Data policy

Reviewer #2: Yes

Reviewer #3: (No Response)

**********

5. Is the manuscript presented in an intelligible fashion and written in standard English?

Reviewer #2: Yes

Reviewer #3: (No Response)

**********

Reviewer #2: The revised manuscript represents a substantial improvement over the previous version. The authors have carefully addressed the reviewers’ comments and have strengthened several key aspects of the study, including the clarity of the study design, the methodological description, the framing of the findings, and the interpretation of the results within the broader literature. Importantly, the revised version now presents a clearer and more coherent central message, emphasizing the gap between individual knowledge and the structural conditions required to effectively prevent HTLV-1 mother-to-child transmission (MTCT). This framing considerably enhances the public health relevance of the manuscript. Overall, the manuscript now reads as a well-structured exploratory study that contributes valuable evidence on HTLV-1 prevention in an under-studied context. The revisions have addressed the main concerns raised in the previous review round, and the current version is substantially stronger both conceptually and methodologically. Major strengths of the revised manuscript are listed below:

1. Clearer methodological framing: The authors now explicitly describe the study as a descriptive, exploratory, and hypothesis-generating cross-sectional study, which appropriately aligns the study objectives, analytical approach, and interpretation of results. This clarification improves methodological transparency and sets appropriate expectations regarding inference. The explanation of the sample size rationale and convenience sampling is also clearer and more consistent with the exploratory nature of the study.

2. Improved transparency of the analytical approach: The description of the qualitative thematic analysis has been strengthened. The revised text now explains the coding process and the development of themes, which improves the reproducibility and transparency of the qualitative component. Similarly, the inclusion of exploratory comparisons according to time since HTLV-1 diagnosis adds useful analytical depth while appropriately maintaining a cautious interpretation.

3. Stronger conceptual framing of the findings: One of the most important improvements in the revised manuscript is the clearer articulation of the structural barrier represented by postpartum diagnosis for HTLV-1 MTCT prevention. The discussion effectively highlights the contrast between: relatively high knowledge and favorable attitudes among participants, and the health-system limitations that prevent timely diagnosis and effective prevention. This framing significantly enhances the manuscript’s contribution to the global health literature.

4. Better integration with the international literature: The revised discussion now situates the findings within the broader context of HTLV-1 prevention efforts in other endemic settings, including comparisons with studies conducted in French Guiana and Jamaica. These additions strengthen the external relevance of the study and help contextualize the findings within ongoing discussions about screening strategies and breastfeeding recommendations.

5. Appropriate and well-structured limitations section: The limitations are now clearly and appropriately discussed. In particular, the authors acknowledge: the selection bias inherent to studying women attending a specialized urban referral center, the relatively small sample size and convenience sampling, and the limited depth of the qualitative component. This transparent discussion of limitations strengthens the credibility of the study and appropriately bounds the interpretation of the findings.

Minor comment: A small amount of repetition remains in the discussion regarding postpartum diagnosis as a barrier to prevention. Minor editorial streamlining could improve conciseness. These comments are minor and do not affect the overall quality or interpretability of the manuscript.

Reviewer #3: This manuscript presents an important contribution to the understanding of Human T-lymphotropic virus type 1 (HTLV-1) mother-to-child transmission (MTCT) in Peru. By exploring women’s knowledge, attitudes, and practices (KAP), the study provides valuable insights into the sociocultural, clinical, and health system factors influencing HTLV-1 prevention. Given the limited literature on HTLV-1 in Latin America and the public health relevance of vertical transmission, this work has significant implications for policy development, antenatal screening strategies, and targeted educational interventions.

The manuscript is well structured, clearly written, and addresses a critical gap in the field. The integration of quantitative and qualitative findings strengthens its contribution and aligns with the scope of PLOS Global Public Health. With minor revisions, the manuscript will be further enhanced in terms of clarity, rigor, and impact.

Recommendations for Improvement

1. The descriptive statistical approach is appropriate. The authors may consider reporting 95% confidence intervals for key proportions to provide additional context regarding the precision of the estimates.

2. Clarify the Description of the Qualitative Analysis

The qualitative component is based on a single open-ended question and appropriately complements the quantitative findings. To enhance methodological transparency, the authors may briefly describe how responses were grouped into themes and summarized. A concise explanation of how themes were identified would improve clarity without requiring extensive qualitative detail.

3. Clarify Survey Development and Validation

The authors note that the questionnaire was developed by HTLV experts and piloted prior to data collection. Providing additional detail on its development (such as its basis in prior studies, pilot testing procedures, and any steps taken to ensure clarity and content validity) would strengthen the methodological rigor of the manuscript.

4. Minor Editorial Suggestions

The limitations section appropriately acknowledges selection bias. Expanding this discussion to address implications for rural and underserved populations in Peru would further enhance the manuscript’s public health relevance.

Ensure consistency in terminology throughout the manuscript. After defining ‘mother-to-child transmission (MTCT)’ at first use, the abbreviation should be used consistently in the main text to enhance clarity and readability.

**********

what does this mean?). If published, this will include your full peer review and any attached files.

**Do you want your identity to be public for this peer review?**  If you choose “no”, your identity will remain anonymous but your review may still be made public.

For information about this choice, including consent withdrawal, please see our Privacy Policy

Reviewer #2: **Yes:**  Angelica E Miranda

Reviewer #3: **Yes:**  Ramona Moles

**********

[NOTE: If reviewer comments were submitted as an attachment file, they will be attached to this email and accessible via the submission site. Please log into your account, locate the manuscript record, and check for the action link "View Attachments". If this link does not appear, there are no attachment files.]

# Author comment: Author response to Decision Letter 2

**DOI:** 10.1371/journal.pgph.0007103.r004

28 Apr 2026

Attachment Submitted filename: Response_PLOS_04_detailed.docx

# Peer review: Decision Letter 2

**DOI:** 10.1371/journal.pgph.0007103.r005

**Authors:** Emma Campbell

28 May 2026

PGPH-D-25-03721R2

Women’s knowledge, attitudes and practices related to mother-to-child transmission of HTLV-1 in Peru

PLOS Global Public Health

Dear Dr. Gotuzzo,

Thank you for submitting your manuscript to PLOS Global Public Health. After careful consideration, we feel that it has merit but does not fully meet PLOS Global Public Health’s publication criteria as it currently stands. Therefore, we invite you to submit a revised version of the manuscript that addresses the points raised during the review process.

The revised manuscript has been assessed and there are still elements that need to be improved before publication can be considered. Specifically the reviewer has noted that additional detail is required regarding the qualitative analysis, the validation and pilot testing of the questionnaire and the policy implications of the generated results. Please review their comments below and make the appropriate revisions.

Please submit your revised manuscript by Jul 26 2026 11:59PM. If you will need more time than this to complete your revisions, please reply to this message or contact the journal office at globalpubhealth@plos.org. When you're ready to submit your revision, log on to https://www.editorialmanager.com/pgph/ and select the 'Submissions Needing Revision' folder to locate your manuscript file.

Please include the following items when submitting your revised manuscript:

- A letter that responds to each point raised by the editor and reviewer(s). You should upload this letter as a separate file labeled 'Response to Reviewers'.
- A marked-up copy of your manuscript that highlights changes made to the original version. You should upload this as a separate file labeled 'Revised Manuscript with Track Changes'.
- An unmarked version of your revised paper without tracked changes. You should upload this as a separate file labeled 'Manuscript'.

Guidelines for resubmitting your figure files are available below the reviewer comments at the end of this letter.

As the corresponding author, your ORCID iD is verified in the submission system and will appear in the published article. PLOS supports the use of ORCID, and we encourage all coauthors to register for an ORCID iD and use it as well. Please encourage your coauthors to verify their ORCID iD within the submission system before final acceptance, as unverified ORCID iDs will not appear in the published article. *Only*  the individual author can complete the verification step; PLOS staff *cannot*  verify ORCID iDs on behalf of authors.

We look forward to receiving your revised manuscript.

Kind regards,

Emma Campbell, Ph.D

Staff Editor

PLOS Global Public Health

Journal Requirements:

If the reviewer comments include a recommendation to cite specific previously published works, please review and evaluate these publications to determine whether they are relevant and should be cited. There is no requirement to cite these works unless the editor has indicated otherwise.

Additional Editor Comments (if provided):

[Note: HTML markup is below. Please do not edit.]

Reviewers' comments:

Reviewer's Responses to Questions

**Comments to the Author**

Reviewer #3: All comments have been addressed

Reviewer #4: (No Response)

**********

publication criteria?

Reviewer #3: Yes

Reviewer #4: Yes

**********

3. Has the statistical analysis been performed appropriately and rigorously?

Reviewer #3: Yes

Reviewer #4: N/A

**********

4. Have the authors made all data underlying the findings in their manuscript fully available (please refer to the Data Availability Statement at the start of the manuscript PDF file)?

The PLOS Data policy

Reviewer #3: Yes

Reviewer #4: Yes

**********

5. Is the manuscript presented in an intelligible fashion and written in standard English?

Reviewer #3: Yes

Reviewer #4: Yes

**********

Reviewer #3: (No Response)

Reviewer #4: General Assessment

This manuscript addresses an important and understudied public health issue: mother-to-child transmission (MTCT) of HTLV-1 in Peru. The study provides valuable insights into women’s knowledge, attitudes, and practices, particularly within an urban, care-engaged population.

The manuscript is generally well structured, clearly written, and methodologically appropriate for an exploratory cross-sectional study. The integration of quantitative and qualitative findings is a notable strength, contributing to a more comprehensive understanding of the topic.

However, several aspects require clarification or refinement to improve methodological transparency, interpretability, and overall impact.

Comments

1. Qualitative Analysis Description

The qualitative component adds value; however, the description of the analytical process remains limited.

Recommendation:

Please provide a clearer description of:

- the coding processes.

- how themes and subthemes were identified.

- how consistency between coders was ensured.

This will enhance methodological transparency and reproducibility.

2. Survey Development and Validation

The manuscript mentions that the instrument was developed by experts and piloted, but details are limited.

Recommendation:

Consider elaborating on:

- the stepwise development of the questionnaire.

- how prior studies informed item selection.

- the pilot testing process and resulting modifications.

- any procedures used to ensure content validity and clarity.

3. Policy and Implementation Implications

The manuscript highlights prenatal screening as a potential intervention but provides limited discussion of operational feasibility.

Recommendation:

Expand on:

- implementation challenges.

- resource requirements (e.g., water access, formula provision).

- ethical and cultural considerations.

- integration into existing maternal health programs.

Conclusion

This manuscript provides a relevant contribution to the field of HTLV-1 prevention and maternal-child health. With revisions addressing the points above, it has the potential to make a meaningful contribution to the literature.

Recommendation:

Minor revision

**********

what does this mean?). If published, this will include your full peer review and any attached files.

**Do you want your identity to be public for this peer review?**  If you choose “no”, your identity will remain anonymous but your review may still be made public.

For information about this choice, including consent withdrawal, please see our Privacy Policy

Reviewer #3: **Yes:**  Ramona Moles

Reviewer #4: No

**********

[NOTE: If reviewer comments were submitted as an attachment file, they will be attached to this email and accessible via the submission site. Please log into your account, locate the manuscript record, and check for the action link "View Attachments". If this link does not appear, there are no attachment files.]

# Author comment: Author response to Decision Letter 3

**DOI:** 10.1371/journal.pgph.0007103.r006

5 Jun 2026

Attachment Submitted filename: Response_PLOS_05_detailed.docx

# Peer review: Decision Letter 3

**DOI:** 10.1371/journal.pgph.0007103.r007

**Authors:** M. A. Rifat

30 Jun 2026

PGPH-D-25-03721R3

Women’s knowledge, attitudes and practices related to mother-to-child transmission of HTLV-1 in Peru

PLOS Global Public Health

Dear Dr. Eduardo Gotuzzo,

Thank you for submitting your manuscript to PLOS Global Public Health. After careful consideration and based on review from another reviewer, we feel that your manuscript has merit but still require a revision for fully meeting the PLOS Global Public Health’s publication criteria. Therefore, we invite you to submit a revised version of the manuscript that addresses the points raised during the review process. If you disagree with any of the comments from the reviewer, please explain in rebuttal letter.

Please submit your revised manuscript by 30 July 2026. If you will need more time than this to complete your revisions, please reply to this message or contact the journal office at globalpubhealth@plos.org. When you're ready to submit your revision, log on to https://www.editorialmanager.com/pgph/ and select the 'Submissions Needing Revision' folder to locate your manuscript file.

Please include the following items when submitting your revised manuscript:

- A letter that responds to each point raised by the editor and reviewer(s). You should upload this letter as a separate file labeled 'Response to Reviewers'.
- A marked-up copy of your manuscript that highlights changes made to the original version. You should upload this as a separate file labeled 'Revised Manuscript with Track Changes'.
- An unmarked version of your revised paper without tracked changes. You should upload this as a separate file labeled 'Manuscript'.

Guidelines for resubmitting your figure files are available below the reviewer comments at the end of this letter.

As the corresponding author, your ORCID iD is verified in the submission system and will appear in the published article. PLOS supports the use of ORCID, and we encourage all coauthors to register for an ORCID iD and use it as well. Please encourage your coauthors to verify their ORCID iD within the submission system before final acceptance, as unverified ORCID iDs will not appear in the published article. *Only*  the individual author can complete the verification step; PLOS staff *cannot*  verify ORCID iDs on behalf of authors.

We look forward to receiving your revised manuscript.

Kind regards,

M. A. Rifat

Academic Editor

PLOS Global Public Health

**Journal Requirements:**

If the reviewer comments include a recommendation to cite specific previously published works, please review and evaluate these publications to determine whether they are relevant and should be cited. There is no requirement to cite these works unless the editor has indicated otherwise.

Please review your reference list to ensure that it is complete and correct. If you have cited papers that have been retracted, please include the rationale for doing so in the manuscript text, or remove these references and replace them with relevant current references. Any changes to the reference list should be mentioned in the rebuttal letter that accompanies your revised manuscript. If you need to cite a retracted article, indicate the article’s retracted status in the References list and also include a citation and full reference for the retraction notice.

**Additional Editor Comments (if provided):**

[Note: HTML markup is below. Please do not edit.]

Reviewers' comments:

Reviewer's Responses to Questions

**Comments to the Author**

Reviewer #3: All comments have been addressed

Reviewer #4: All comments have been addressed

Reviewer #5: (No Response)

**********

publication criteria?

Reviewer #3: Yes

Reviewer #4: Yes

Reviewer #5: Partly

**********

3. Has the statistical analysis been performed appropriately and rigorously?

Reviewer #3: Yes

Reviewer #4: N/A

Reviewer #5: No

**********

4. Have the authors made all data underlying the findings in their manuscript fully available (please refer to the Data Availability Statement at the start of the manuscript PDF file)?

The PLOS Data policy

Reviewer #3: Yes

Reviewer #4: Yes

Reviewer #5: No

**********

5. Is the manuscript presented in an intelligible fashion and written in standard English?

Reviewer #3: Yes

Reviewer #4: Yes

Reviewer #5: Yes

**********

**Reviewer #3:**  The authors have adequately addressed the reviewers' concerns

**Reviewer #4** : Reviewer’s Comments

I have reviewed the revised version of the manuscript entitled “Women’s knowledge, attitudes and practices related to mother-to-child transmission of HTLV-1 in Peru.” The authors have satisfactorily addressed the comments raised during the previous rounds of review.

The manuscript has been substantially improved. The description of the qualitative analysis is now clearer and more transparent, and the process of survey development and piloting has been better detailed. In addition, the discussion has been strengthened, particularly regarding the policy and implementation implications of the findings.

This study addresses an important and under-researched public health issue. The findings provide relevant insights into barriers to HTLV-1 mother-to-child transmission prevention in endemic settings and are likely to be of interest to the readership of PLOS Global Public Health. The study limitations are appropriately acknowledged, and the conclusions are well supported by the data.

Overall, the manuscript meets the standards for publication in this journal.

Recommendation: Accept

**Reviewer #5:**  Review date: 30 JUNE 2026

Comments or suggestions for authors

Abstract

1. The suggested title would be “Women’s knowledge, attitude and practices related to transmission of Human T-Cell Lymphotropic Virus Type-1 through breastfeeding in Peru”. [ Explanation: Better to avoid any short forms in the title; To make the title easy and readers understandable, only knowledge assessment can give insights about attitude and practice. However, authors can subsequently discuss about attitude and practice based on assessing the knowledge]

2. Can it cause leukemia or other diseases? If so, please start with…” …can be transmitted from mother to child through breastfeeding which can cause leukemia or other serious diseases”. [ Explanation: Readers are interested to know the effect of virus]

3. Lack access to safe water is not direct cause of transmission. Hence, it is recommended to omit such words in the abstract, if irrelevant.

4. Recommended to avoid “…many population”. [Explanation: “many” is a vague term, not suitable in the abstract].

5. Small sample size and inadequate statistics. Please, mention some basic socio-demographic status of the respondents such as education [e.g. % of respondents were post-graduate/graduate/completed primary school education], occupation [e.g. % of respondents had jobs, business, housewife and others], marital status etc.

6. Please explain why water and swage services are associated with transmission of HTLV-1 as it the HTLV-1 transmit through blood, breastmilk and sexual intercourse.

7. The higher rates [e.g. more than 85%] of knowledge on three core routes of transmission is surprising for such rare virus transmission. Please check and justify.

8. Please capture the source of knowledge and rates of knowledge sources [e.g. breastfeeding counseling, media advertisement/health education/billboard/mobile SMS/social media]

9. Please clearly state and rate the practice related stigma [e.g. formula-feed]. It is recommended to describe the rate instead of number of respondents.

Introduction

1. Line number 50, please avoid insertion of RR, CI with references. It is recommended to insert all references at the end of the sentence. Authors are suggested to review more articles to shape citation style.

2. Line number 57-58, recommended to insert reference [10] at the end of the sentence. Similarly, for other sentences. In addition, recommended to use recent citation instead of old references.

3. From line number 63, recommended to start explaining about knowledge, attitude and practice related to HTLV-1 transmission.

4. The line number from 64 to 73, authors nicely mentioned the interventions in Japan, its feasibility in low-income settings and emphasis on strategic balance of interventions based on socio-demographic and socio-cultural factors. Please also discuss about knowledge and practice related to maternal and child transmission of HTLV-1. Please explain what level of knowledge the researchers found in different settings [ e.g. % mothers had higher and ---% of mother had average knowledge, cut-off point for higher, average and below level knowledge related to MTCT, also discuss which occupation group or income groups had higher knowledge, urban-rural difference – if any].

5. The line number 75-76, authors mentioned higher prevalence of HTLV-1 than other major diseases. It is recommended to insert the percentage of each disease. In line 76, please revise the sentence as past form [ e.g. “was” instead of “is”].

6. In line number 76-78, prenatal screening is cost-effective to prevent transmission of HTLV-1; Please also discuss about knowledge sources [e.g. screening, social media and or mass media advertisement, community sessions].

Methods

1. In the line 95, please omit the names of doctors or clinicians to ensure anonymization. According to Helsinki declaration, it is necessary to avoid any names. You can acknowledge their contribution in acknowledgement section.

2. In the line number 99, elaborate the short form of IMTAVH and the line number 97-98 can be inserted after the line number 99. In line 98, please mention the number of participants you have included. The age, occupation, income, residence, history of exposure to HTLV-1, co-morbidity, inclusion and exclusion criteria and other information required for this study.

3. In the line number 114, elaborate the short forms of FG, AP and PC.

4. The sentence from 120-122, need improvement to make it understandable.

5. The line number 126-130, information can be replaced with above mentioned area.

6. In the line 150, authors estimated 50 samples. Please clearly state the actual number of enrolled participants.

7. In the line number 151, authors mentioned funding information. It is recommended to exclude funding information in methods section. It is necessary to mention separately.

8. Please mention the scientific steps of participant’s enrollment or a sub-heading of consent to participate such as sharing of study objectives, anonymization, risks and benefits, tentative time of interview, methods of interview (e.g. face to face or other process), freedom of acceptance or rejection and other points of Helsinki declaration. Then mention whether you received a written informed consent.

Results

1. In table 1., Huge correction required. The baseline information should be age [e.g. age range – 18-45year, 46-60 year], education, occupation, income, residence, religion, smoking and diabetes status, and other socio-demographic information [e.g. marital status]. CI range and 95% of CI wrongly presented which is not essential for table 1. In addition, for the value 1, the CI range 0-12 is usually not acceptable.

2. Line number 182 to 187, need proper clarification.

3. For table 2., authors are requested to learn more about organization of tables utilizing statistical software [e.g. stata], capturing the key facts, how to write CI range.

4. Authors are suggested to group the age range. Was it only two age groups?

Discussion

1. In the line 228, please state how you declared middle- or high-income women.

2. There is scope of improving English throughout the discussion and other parts of the manuscript.

References

1. Recommended to replace old references with recent references.

Overall: The study was highly technical and not properly presented/organized. The statistical analysis and organization of tables were not adequately inserted with lack of interpretation.

**********

what does this mean?). If published, this will include your full peer review and any attached files.

**Do you want your identity to be public for this peer review?**  If you choose “no”, your identity will remain anonymous but your review may still be made public.

For information about this choice, including consent withdrawal, please see our Privacy Policy

Reviewer #3: **Yes:**  Ramona Moles

Reviewer #4: No

Reviewer #5: **Yes:**  Dr. Md. Abu Sayem

**********

[NOTE: If reviewer comments were submitted as an attachment file, they will be attached to this email and accessible via the submission site. Please log into your account, locate the manuscript record, and check for the action link "View Attachments". If this link does not appear, there are no attachment files.]

**Figure Resubmissions:**

Attachment Submitted filename: Reviewers comments_Reviewer_3.pdf

# Author comment: Author response to Decision Letter 4

**DOI:** 10.1371/journal.pgph.0007103.r008

22 Jul 2026

Attachment Submitted filename: Response_PLOS_06_detailed.docx

# Editor report: Decision Letter 4

**DOI:** 10.1371/journal.pgph.0007103.r009

**Authors:** M. A. Rifat

28 Jul 2026

Women’s knowledge, attitudes and practices related to mother-to-child transmission of HTLV-1 in Peru

PGPH-D-25-03721R4

Dear Dr. Eduardo Gotuzzo,

We are pleased to inform you that your manuscript 'Women’s knowledge, attitudes and practices related to mother-to-child transmission of HTLV-1 in Peru' has been provisionally accepted for publication in PLOS Global Public Health.

Before your manuscript can be formally accepted you will need to complete some formatting changes, which you will receive in a follow up email. A member of our team will be in touch with a set of requests.

Please note that your manuscript will not be scheduled for publication until you have made the required changes, so a swift response is appreciated.

IMPORTANT: The editorial review process is now complete. PLOS will only permit corrections to spelling, formatting or significant scientific errors from this point onwards. Requests for major changes, or any which affect the scientific understanding of your work, will cause delays to the publication date of your manuscript.

If your institution or institutions have a press office, please notify them about your upcoming paper to help maximize its impact. If they'll be preparing press materials, please inform our press team as soon as possible -- no later than 48 hours after receiving the formal acceptance. Your manuscript will remain under strict press embargo until 2 pm Eastern Time on the date of publication. For more information, please contact globalpubhealth@plos.org.

Thank you again for supporting Open Access publishing; we are looking forward to publishing your work in PLOS Global Public Health.

Best regards,

M. A. Rifat

Academic Editor

PLOS Global Public Health

***********************************************************

Reviewer Comments (if any, and for reference):
